# Determinants of Child Stunting, Wasting, and Underweight: Evidence from 2017 to 2018 Pakistan Demographic and Health Survey

**DOI:** 10.1155/2023/2845133

**Published:** 2023-03-04

**Authors:** Maryam Siddiqa, Gulzar H. Shah, Tilicia L. Mayo-Gamble, Amber Zubair

**Affiliations:** ^1^Department of Mathematics and Statistics, Room# 228, Maryam Block, Sector H-10 International Islamic University Islamabad, Islamabad, Pakistan; ^2^Department of Health Policy and Community Health, Jiann-Ping Hsu College of Public Health, Georgia Southern University, P.O. Box 8015 Statesboro, GA 30460, Statesboro, Georgia; ^3^Department of Health Policy and Community Health Georgia Southern University, P.O. Box 8015 Statesboro, GA 30460, Statesboro, Georgia; ^4^International Islamic University Islamabad, Islamabad, Pakistan

## Abstract

Child malnutrition persists in low-resource countries such as Pakistan, indicating an urgent need for interventions and policies aimed to address this critical population health issue. The World Health Organization Global Target 2025 includes the reduction of malnourishment in the form of stunting, wasting, and low weight. This study aims to examine the prevalence of factors associated with three measures of child malnutrition, i.e., stunting, wasting, and low weight in Pakistan. This study uses a secondary data analysis design based on data from Pakistan Demographic and Health Survey (2017-18) that used a two-stage cluster sampling approach. National level data covering urban and rural areas were used for this study consisting of 4,226 children less than 5 years of age. Univariate and multivariable analyses using logistic regression models were conducted. Over 23% of the children were underweight, 8.0% suffered wasting, and 37.7% were stunted. Children with small size at birth (<45.7 cm), those who were average in size (45.7 to 60 cm) at birth were less likely to be stunted (AOR, 0.4890) and underweight (AOR, 0.538). Children with large size at birth (>60 cm) were also less likely to be stunted (AOR, 0.288) and underweight (AOR, 0.538). Children who consumed fresh milk were less likely to be classified as wasted (AOR, 0.524) than those children who did not consume fresh milk. The children in high- and middle-economic status families were less likely to be stunted, underweight, or wasted. Children of mothers who had secondary and higher education were less likely to be stunted (AOR, 0.584) and were less likely to be underweight (AOR, 0.668) than illiterate mothers' children. Children of working mothers were less likely to be wasted compared to children of nonworking mothers (AOR, 0.287). Maternal BMI is also inversely associated with being underweight because overweight and obese mothers were less likely to have underweight children (AOR, 0.585). Our findings reflect a need to design targeted public health policies and community-based education that emphasize the mother's education on nutrition health and provide socioeconomic resources that enable mothers to provide dietary needs that prevent malnutrition.

## 1. Introduction

Undernutrition is a persistent global public health issue that contributes to the physical, mental, and cognitive decline of children in low-resource countries [1]. Proper nourishment is necessary for mental and physical development in a child's early years of life [2]. Malnourished children are more likely to die from illnesses such as malaria, diarrhea pneumonia, and measles than children who receive adequate nutrition [3]. The most common forms of undernutrition are protein-energy, malnutrition caused by deficiencies in protein, and malnutrition of energy intake, which results from infection and excessive nutrient loss after child birth or during carrying a child or childbearing [4]. Poverty, low levels of education, presence of infections, low socioeconomic status, and poor access to health services are all factors that contribute to malnourishment in children [4].

The World Health Organization Global Target 2025 includes goals for the reduction of malnourishment in the form of stunting, wasting, and low weight [5]. In 2019, 38.3 million (5.6%) were overweight, 47 million (6.9%) were wasted, and 144 million children were stunted (21.3%) globally. The pervasiveness of malnourished children is higher in underdeveloped countries [6–10]. Fifty percent of undernourished children were located in three countries of South Asia (i.e., India, Bangladesh, and Pakistan) [11]. Worldwide, 27% of stunted children and 17% of wasted children lived in low-income countries [11]. The prevalence rates in Asia during 2019 forwasting, stunting, and overweight in children aged 5 years were 69%, 54%, and 45%, respectively [11]. In South Asian countries, subsequently India, the majority of the reported stunted children belong to Pakistan [12].

The prevalence of child undernutrition is higher in low-income countries [6–10]. Shahid and colleagues note that in low-income countries, the prevalence of stunting increased from 3.8% to 3.97% between the years 2000 and 2019, whereas in economically stable countries, the stunting rates decreased from 2.3% to 1.9% [7]. The highest rates of child undernutrition were found in Africa and South Asia, with 50% of the wasted children residing in Southern Asia. This includes Pakistan, where undernutrition impacts children below 5 years of age [13]. Pakistan has high rates of child mortality (6.93% or 69.3 per 1000) in children below five years, which is nearly double the rates of neighboring countries such as India (3.66%), Nepal (3.22%), and Bhutan (2.97%) [13]. In addition, the National Nutrition Survey (2018) found that in Pakistan, among children below the age of five, 40% were stunted, 17.7% were wasted, and 28.9% were underweight [14].

Previous studies have shown that factors such as short birth interval, maternal BMI, several or numerous births, household wealth index, mother education, and low birth weight are associated with stunted growth [10, 15–20]. In addition, community-level factors such as improved water, hygiene, and sanitation are also significant determinants of stunting [18, 21]. For mothers, safe water source, gender equality, food availability, and education are important factors for reducing stunting [10, 16, 19, 20, 22]. In the existing literature, it is unclear which of the community-level and maternal factors impact children and mothers in Pakistan. The only research that has apparently studied the same topic has masked the subcategories of malnutrition, coding it as a dichotomous variable. In contrast, the current study investigated specific categories of stunting, wasting, and underweight. Therefore, the findings of the current study are much more actionable because the intervention to address three levels of undernutrition, i.e., stunting, wasting, and underweight, needs to be different and customized to the level of malnutrition. For instance, stunting measures chronic deficiencies in nutrition, whereas, wasting status indicates acute nutritional deficiency. Malnutrition is a broader term indicating an imbalanced intake of nutrition. Thus, the purpose of this study is as follows: (1) to examine the extent of child malnutrition in Pakistan, as reflected by stunting, wasting, and underweight and (2) to determine which child characteristics and maternal as well as societal factors available from the secondary data are associated with child malnutrition measures of stunting, wasting, and low weight.

## 2. Materials and Methods

### 2.1. Study Population

This study is based on secondary analysis of the data extracted from a national survey, Pakistan Demographic and Health Survey, version 2017-18 (PKKR71FL file of the data)^2^. In the present study, 15,068 ever-married women from 8 different geographical areas of Pakistan were questioned about the level of child malnutrition. Information about 12,708 children of age less than 5 years is available from the data. The current study investigated the determinants of stunting, wasting, and underweight of under five years children. For this purpose, after cleaning the data, the alive children's total sample size comprised 4,226 children under 5 years of age included in the analysis who have complete information about nutritional variables.

The 2017-18 Pakistan Demographic and Health Survey (PDHS) is a nationally representative survey conducted by the National Institute of Population Studies (NIPS), and the ICF International, United States. The 2017-18 PDHS used a multistage sampling design, comprising 16 strata. Two strata (an urban and a rural) came from each of the 8 geographic territories of Pakistan; Azad Jammu and Kashmir; Islamabad Capital Territory; Gilgit Baltistan (GB); FATA (Federally Administered Tribal Areas); Punjab; Sindh; Khyber Pakhtunkhwa; and Balochistan. A complete list of enumeration blocks (EBs) served as a sampling frame for the first stage. In the 1^st^ stage of sampling, 580 sampling units (the EBs) were selected (295 rural clusters and 285 urban clusters) grounded on probability proportional to size within an EB, of which 561 clusters were successfully surveyed. Systematic sampling with equal probability was employed in the second stage for selecting the 28 households in each cluster that were selected for an interview, resulting in a final sample of 16,240 households [23].

### 2.2. Response Variables-Anthropometric Indicators for Child Malnutrition

The three response variables *o* of this study were the nutritional situation of children, which was intended from weight for age z-score (WAZ), height for age z-score (HAZ), and weight for height z-score (WHZ), considering moderately (−3 to −2 of z-score) to severely malnourished (<−3 of z-score); whereas, a child having greater than −2 of z-score reflected a normal nutritional status. “Height for age” shows linear growth retardation and cumulative growth deficits in children. “Height for age” is prolonged malnutrition which indicates the insufficiencies of nutrient value over an elongated period. Low “height for age” is known as stunting. Reduced “weight for height” is considered acute malnutrition. “Weight for height” measures the body mass compared with body height. “Weight for height” can be measured as an indicator of the recent nutritional standing. Less “weight for height” is referred to as wasting. “Weight for age” measures body mass relative to age. The “weight for age” index is a complex measure of together “weight for height” and “height for age.” “Weight for age” is an indicator of overall malnourishment and takes accounts for acute and chronic undernourishment. Stunted (i.e., moderate and severe), wasted (i.e., moderate and severe), and underweight (i.e., moderate and severe), three response variables are used as binary variables with “NO” category (no stunted, no wasted, and no underweight) being coded as zero while stunted, wasted, and underweight being coded as one.

The anthropometric extent of the nutritious status of a child grounded on height and weight ratios is the most frequently used assessment method, which is to check whether the child is properly nourished or undernourished [4, 15, 17]. These ratios are denoted as height-for-age (stunted), weight-for-height (wasted), and weight-for-age (underweight) *Z*-scores. Observed growth performance is assessed in comparison with a standard that is considered preeminent to exemplify normal growth. The standard that is currently broadly used for the said purpose in several nations is developed by the National Centre for Health Statistics (NCHS) of the USA, which is established on growth measurements of the bulk of American children data [23]. WHO Child Growth Standards represent normal growth by controlling environmental conditions and can be used for growth and development in children worldwide as an international reference since they meet most of the criteria necessary for this purpose [23]. Deficits in each of the anthropometric indicators are reflected as a sign of malnutrition and indicated as wasting, stunting, and underweight. In general, the proportion of children falls less than −2 standard deviations of *z*-score and below the median of the international reference population from WHO reference growth standards is used to determine the prevalence of undernutrition in children [2, 23].

### 2.3. Independent Variables

Independent variables considered in this study are child, maternal, and sociodemographic characteristics of children below 5 years of age; more details of these independent variables are presented in Table 1 along with their categories and frequencies.

### 2.4. Statistical Analysis

Descriptive analysis of children, their sociodemographic, and maternal characteristics were reported in terms of frequencies of variables. Unadjusted associations were tested using the bivariate logistic regression. To check the assumptions of multicollinearity, the diagnostic test VIF (variance inflation factor) was used. To detect outliers, Cook's D plot was used. The multivariate logistic regression analysis was performed to determine the factors associated with malnutrition anthropometric measures. A binary logistic regression was employed to investigate the risk factors of three separate binary dependent variables with several independent variables by using the stepwise backward elimination method. All statistical analyses were conducted using STATA (Version 13) [24].

## 3. Results

### 3.1. Descriptive Statistics

**Figure 1:**
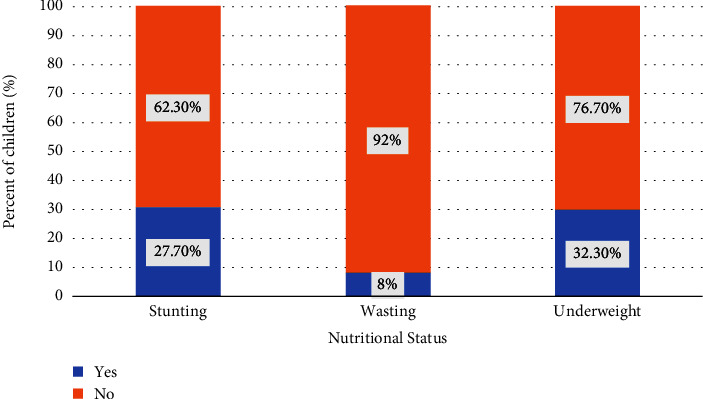
Nutritional status of children aged 5 years or younger in Pakistan.

**Figure 2:**
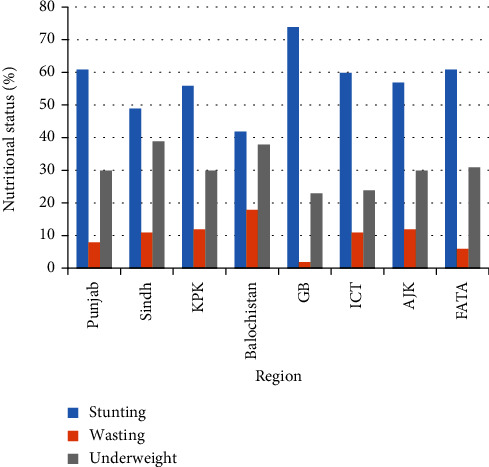
Nutritional status of children aged 5 years or younger in 8 regions of Pakistan.

**Figure 3:**
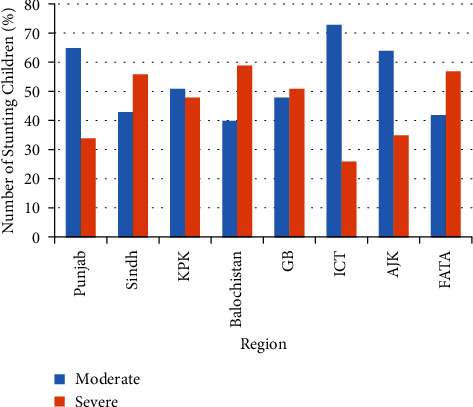
Percent of stunted-growth children aged 5 years or younger in regions of Pakistan.

**Figure 4:**
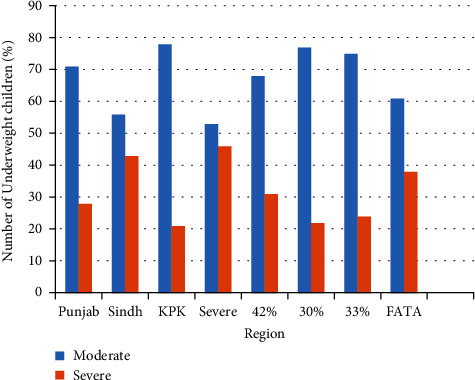
Percent of underweight children aged 5 years or younger in regions of Pakistan.

**Figure 5:**
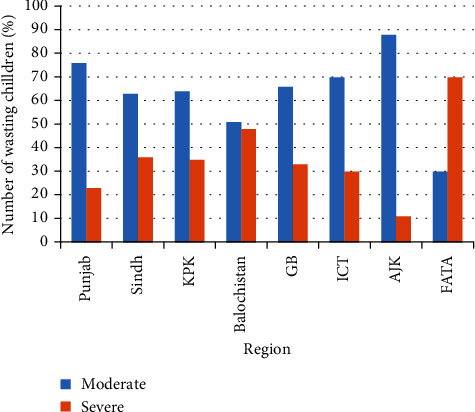
Percent of wasted children aged 5 years or younger in regions of Pakistan.

Figure 1 shows the nutritional status according to three anthropometric measures of children aged 5 years or younger in Pakistan. The percentage of children reported to have stunted growth was 37.7%. Roughly, one-in-four (23.3%) children were underweight and those with a “wasting” status were 8.0%. Figures 2–5 present the prevalence of stunting, wasting, and underweight by regions of Pakistan.

Table 1 represents descriptive statistics for societal, maternal, and child-level characteristics. Around half of the children (49.2%) were females and the majority of children (75.3%) perceived average size at birth. The majority of children (68.3%) did not consume fresh milk and (75.5%) did not obtain a postnatal checkup. Also, around half (57.3%) of the children were informed of being breastfed and in half of the children (50.7%), breastfeeding was commenced immediately after birth. More than half of the mothers (51.5%) had no formal education, 10.9% were underweight, and 48.4% were overweight or obese. A small section of mothers (15.4%) were formally employed whereas 84.6% were not employed in paid jobs. Women were mostly nonsmokers (96.5%). Among societal factors, 56.8% of the children were members of families residing in rural areas. The majority of the children belonged to families who had “improved” toilet facilities (76.7%) and improved water sources (85.4%). Nearly, 46.3% of the children were from poor families and 56.7% of the children belonged to educated fathers.

Table 2 indicates that no multicollinearity problem exists in the regression analysis as all independent variables' VIF values lie under 3.

**Figure 6:**
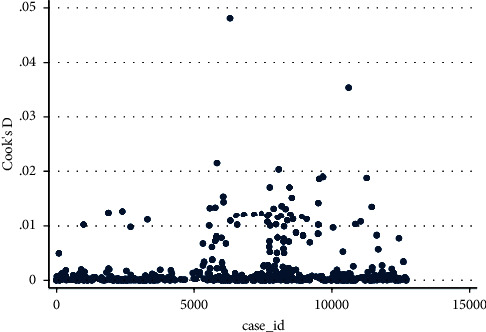
Graph showing Cook's D vs. case ID.

Cook's D graph attained by plotting probabilities versus case IDs to examine for outliers in data is shown in Figure 6. Table 2 specifies that no observation exceeds the cut-off criteria. Only a few observations lie distantly from the actual body of data, but in fact, these observations do not lie outside the limit.

### 3.2. Multivariate Logistic Regression Analysis

#### 3.2.1. Child Characteristics

Results of the multivariable logistic models (Table 3) show that the age of a child is positively associated with stunting. Children aged 13–24 months (AOR, 2.481; 95% CI, 1.714–3.593), 25–36 (AOR, 3.823; 95% CI, 2.637–5.543), 37–48 (AOR, 3.435; 95% CI, 2.306–5.114), and 49–60 (AOR, 3.194; 95% CI, 2.049–4.978) months are more probable to be stunted as compared to children aged 0–6 months. Child birth size was contrariwise associated equally with stunting and underweight. Children who were of an average birth size are less likely to be stunted (AOR, 0.489; 95% CI, 0.381–0.628) and underweight (AOR, 0.538; 95% CI, 0.413–0.701) compared to those of a small size at birth, while children of a larger size at birth are also less likely to be stunted (AOR, 0.288; 95% CI, 0.170–0.486) and underweight (AOR, 0.286; 95% CI, 0.152–0.537) than children of a small birth size. Female children were less likely to be stunted compared to male children (AOR, 0.751; 95% CI, 0.608–0.929). Children who consumed fresh milk were less likely to be classified as wasting (AOR, 0.514; 95% CI: 0.319–0.879) than those who had not consumed fresh milk.

#### 3.2.2. Maternal Characteristics

Children of those mothers who consumed iron tablets during pregnancy were less likely to be stunted (AOR, 0.795; 95% CI, 0.637–0.992) than those mothers who did not take iron supplements during pregnancy. Mother's education was inversely associated with both stunting and being underweight. Children belonging to more educated mothers were less likely to be stunted (AOR, 0.584; 95% CI: 0.424–0.806) and were less likely of being underweight (AOR, 0.668; 95% CI: 0.459–0.973) than illiterate mothers' children. Children of working mothers were less likely to be wasted compared to children of nonworking mothers (AOR, 0.277; 95% CI: 0.114–0.781). Maternal BMI is also inversely associated with being underweight. The result indicates that children of obese and overweight mothers are less likely to be underweight (AOR, 0.585; 95% CI: 0.403–0.850) than underweight mothers' children.

#### 3.2.3. Socioeconomics Characteristics

The children belonging to middle economic status families were less likely to be stunted (AOR, 0.559; 95% CI, 0.4030–0.775), less likely to be underweight (AOR, 0.462; 95% CI, 0.317–0.673), and less likely to be wasted (AOR, 0.351; 95% CI, 0.172–0.716). Children from families with higher incomes were less likely to suffer stunting (AOR, 0.555; 95% CI, 0.404–0.763) and wasting (AOR, 0.521; 95% CI, 0.317–0.855) and being underweight (AOR, 0.398; 95% CI, 0.271–0.583) compared to children of poor families. Children who belonged to families having access to improved water sources were less likely to be stunted (AOR, 0.734; 95% CI: 0.554–0.972) and were less likely of being underweight (AOR, 0.645; 95% CI: 0.484–0.860) compared to their counterparts. Children who belonged to families that resided in Sindh and Baluchistan are more likely to be stunted and underweight among all the regions of Pakistan. The results also indicate that children living in Sindh (AOR, 2.23; 95% CI: 1.570–3.170) and Baluchistan (AOR, 1.777; 95% CI: 1.212–2.604), and FATA (AOR, 2.147; 95% CI: 1.4162–3.318) were more likely of being stunted. Furthermore, children located in Sindh (AOR, 3.349; 95% CI: 2.275–4.929) and Baluchistan (AOR, 3.454; 95% CI: 2.288–5.214) have higher odds of being underweight.

## 4. Discussion

In Pakistan, child malnutrition is a severe health problem that contributes to the risk of morbidity and infant mortality. Our study demonstrates that child birth size, child age, intake of fresh milk, gender of child, mother's education, mother's working status, maternal BMI, iron supplement intake during pregnancy, economic status, source of water, and some region locality (Sindh and Baluchistan) are all significant contributors to malnutrition among children in Pakistan.

Our study found that the risk of stunting increases as the child's age increases. This finding is consistent with the findings of previous literature, which reported that children less than one year are less probable to be malnourished [15–18, 20, 22, 25–39]. With growing age, caloric intake is required from diverse food groups. Poor families with limited resources may not be able to fulfill the dietary needs of growing children. As a result, the child's growth is stunted. One additional potential explanation may be that toddlers get less care and attention from parents in comparison to infants. One study suggests that mothers' attention focuses on the dietary needs of younger children as they need more care [40]. In addition, food and medical care experiences are higher for older children and as a result may be sacrificed to care for younger children.

In our study, as compared to male children, female children were less likely to be stunted. This finding is consistent with the literature [3, 10, 15–17, 36, 41–45]. However, there are studies conducted in Pakistan, Ethiopia, and Nepal that concluded that females had a higher risk of stunting as compared to males [21, 39, 46]. Boys were more susceptible to malnutrition than girls because they require relatively more calorie-dense food for proper growth [47, 48]. According to the Pakistan Dietary Recommendations, boys aged 10–17 require more protein, iron, and zinc than girls [48]. The guidelines also state that boys in this age category, while having comparable body weight, burn more energy than girls [48].

Our study showed that the variable child's birth size was inversely associated with their nutrition status underweight and stunting. Children that were small at birth had a greater risk of being underweight and stunted as compared to children perceived as average or large birth size. These findings are consistent with the findings of many previous studies [4, 11, 15, 17, 21, 31, 44–46], which suggest that children with small birth sizes were more probable to be malnourished. Small birth size may be caused by insufficient maternal nutrition during pregnancy, a time when the child is totally reliant on the mother for nutrition. Any maternal nutritional deficiency affects the growth and development of the foetus. Likewise, mothers who took iron supplements during pregnancy had a lower risk of stunted children. This is consistent with the findings by Tariq et al. reference [18] who reported that children whose mothers did not take the iron supplement during pregnancy were more probable to be stunted.

Our finding that children of obese and overweight mothers are less likely to be underweight is an important consideration. This is perhaps so because households with an abundance of foods may make parents obese but reduce the risk of malnutrition among children as malnutrition includes undernutrition (wasting, stunting, and underweight), inadequate vitamins or minerals, overweight, and obesity. Similar results were found in the literature [4, 10, 15, 17, 18, 46, 49] revealing that children belonging to underweight mothers were more probable to be malnourished. The lower economic profile could be the major reason for poor nutritional status among mothers due to limited access to nutrition-rich diets for both the mother and child. Children of working mothers also had a lower risk of wasting compared to children of nonworking mothers. This finding is consistent with a study by Khan et al. [20] that suggests the nutritional status of children is positively impacted by the mothers' employment level.

There were other maternal factors, such as the women's education, that were significant. The role of women's education can lead to multiple benefits, including a better thoughtfulness of health beliefs, cognitively stimulating activities of children, securing higher incomes, and consequently better opportunities towards higher standards of living [50]. Educated mothers are more inclined to make sure that their children have a nutritive diet, acquire proper healthcare with well-timed vaccination, and are raised as healthy adults [51]. Better-educated societies can overcome poverty and improve the overall health status [52]. Media campaigns for public awareness regarding complementary feeding regimes, vital vitamin-rich foods, and primary nutrients groups, would be helpful to reinforce its significance.

### 4.1. Further Recommendations and Suggestions

Our study suggests that the nutritional status of children is significantly influenced by the education level of mothers. Compared to children born to illiterate mothers, children who belonged to educated mothers were less likely to be stunted and underweight. Our research supports the descriptions of the past studies [3, 10, 15–17, 21, 34, 37, 47, 53–55] that have reported a protective influence of maternal education on children's malnutrition. This may be due to better job opportunities for mothers with higher education. In addition, educated mothers may be more conscious about their children's health and may have adequate healthcare information and means to take care of their children in healthier ways.

In emerging regions of the world like Pakistan, schools can be employed as a platform to educate people about the nutritional value of food by including the relevant material in the syllabus. Awareness about improving nutritional practices and realistic information about making a healthy meal are crucial to stop the cultural and social taboos, particularly for newly pregnant women, to opt for the best food for themselves and their child's health during the perinatal period and early childhood years. In such countries, over half of the people are residents of rural areas, relying exclusively on their crops' production for their meals, which are not satisfactorily diverse to have all essential dietary food groups. In these areas, it is essential to implement interventions to train young mothers and pregnant women about the importance of micronutrients, vitamins, and protein by adding various local, economical vegetables, and proteins into their everyday diet, for enriched nourishment, and healthy living. To improve health, families in rural areas should pick up ways to cultivate nutrient-rich vegetables for instance, leafy greens, beans, and sweet potatoes for iron, protein, and vitamin A, respectively, to duo with chapati and rice-based meals that are staples of daily meals. Pakistan's Government strives to reduce child stunting and take initiatives to control malnutrition. Government bodies launched Ehsaas Nashonuma Programme in 14 districts which provided cash grants of PKR 1,500 for each boy and PKR 2,000 for each girl. Beyond just cash transfers, this program included healthcare for pregnant women and children and facilitated specified nutritious food to pregnant women and children in the most vulnerable locations with the highest childhood stunting rates [56]. Such governmental interventions to reduce malnourishment in mothers and children are more required.

### 4.2. Limitations of This Study

The findings of this study must be viewed within several limitations inherent in the data and methods. First, the study used a cross-sectional survey not allowing the lapse time between the predictors and the outcome variables to be included in the study. Therefore, the relationships tested in the study must be treated merely as associations and no causal inferences should be drawn. Second, the study used secondary data, limiting the selection of the independent variables to the ones available through this source. Regardless of these limitations, this study provides critical evidence that is nationally generalizable and applicable to important policy and public health practice initiatives.

## 5. Conclusions

This study showed that malnutrition is highly prevalent among children in Pakistan and significant disparities exist by the sociodemographic characteristics of children and mothers. These findings have important implications for policies and interventions to curb the high prevalence of child malnutrition and systematically address this serious public health crisis in Pakistan. This persistent public health problem in Pakistan may benefit from targeted approaches to reverse the impact on children. Findings from our study suggest that public health policies and community-level interventions should focus on empowering and supporting mothers. Higher levels of maternal education, household income, and proper nutrition had a protective effect on stunting, wasting, and being underweight among children ages five and under. These findings also have important implications for improving maternal health via awareness campaigns and educating parents on child development and micronutrient supplement intake for a healthy life. Focus on this group also aligns with the global nutrition target of WHO for 2025 to reduce rates of stunting, wasting, and low birth rate and also to improve maternal rates of anemia and breastfeeding.

## Figures and Tables

**Table 1:** Societal, maternal, and child-related characteristics: PDHS (2017-2018).

Variables	Variable attributes	Frequency	Percentage
*Child-level characteristics*			
Child sex	Male	2145	50.8
	Female	2081	49.2

Child age (months)	0–6	548	13
	7–12	405	9.58
	13–24	798	18.9
	25–36	863	20.4
	37–48	853	20.2
	49–60	759	18

Child birth size	Small (<45.7 cm)	725	17.2
	Average (45.7 to 60 cm)	3183	75.3
	Large (>60 cm)	317	7.50

Birth order number	1^st^ born	1046	24.8
	2^nd^–4^th^	2137	50.6
	>5	1043	24.7

Postnatal checkup	No	3051	75.5
	Yes	991	24.5

Fresh milk consumption	No	2847	68.3
	Yes	1323	31.7

Consumed formula milk	No	3118	73.8
	Yes	1106	26.3

Breast feeding	No	1804	42.7
	Yes	2422	57.3

Breastfeeding initiation	Instantly	2052	50.7
	Within 1^st^ hour	958	23.7
	Within 1 day	1040	25.7

*Maternal characteristics*			
Education of mother	No education	2175	51.5
	Primary	579	13.7
	Secondary and higher	1472	34.8

Mother's age	15–19	122	2.89
	20–34	3273	77.4
	35–49	831	19.7

Maternal BMI	Underweight	461	10.9
	Normal	1719	40.7
	Overweight and obese	2046	48.4

Mother's employment	Not employed	3575	84.6
	Employed	651	15.4

Iron tablets' consumption during pregnancy	No	1583	37.5
	Yes	2635	62.5

Cigarette smoking	No	3079	92.6
	Yes	245	7.4

*Socioeconomics characteristics*			
Geographical area	Punjab	906	21.4
	Sindh	806	19.1
	KPK	702	16.6
	Baluchistan	513	12.1
	GB	278	6.58
	ICT	234	5.54
	AJK	436	10.3
	FATA	351	8.3

Type of residence	Urbanicity	1826	43.2
	Rurality	2400	56.8

Toilet facility	No facility	482	11.4
	Not improved	502	11.9
	Improved	3242	76.7

Economic status	Poor	1956	46.3
	Middle	805	19.0
	Rich	1465	34.7

Father's education	No education	1157	27.4
	Primary	620	14.7
	Secondary and higher	2397	56.7

Water source	Unimproved	616	14.6
	Improved	3610	85.4

KPK, Khyber Pakhtunkhwa; GB, Gilgit Baltistan; ICT, Islamabad Capital Territory; AJK, Azad Jammu and Kashmir; FATA, Federally Administered Tribal Area.

**Table 2:** Identification of multicollinearity for probable predictors of malnutrition.

Variables	VIF
Economic status	2.01
Education of mother	1.80
Education of father	1.37
Birth order number	1.33
Fresh milk consumption	1.52
Breastfed	1.28
Mother's age	1.49
Type of residence	1.25
Toilet facility	1.39
Iron tablets' consumption during pregnancy	1.31
Age of child (months)	1.11
Maternal BMI	1.51
Geographical area	1.51
Postnatal checkup	1.23
Breastfeeding initiation	1.20
Consumed formula milk	1.19
Child birth size	1.18
Mother's employment	1.17
Cigarette smoking	1.17
Child sex	1.14
Mean VIF	1.29

**Table 3:** Multivariate logistic regression model for features associated with wasting, stunting, and underweight for children below five years of age PDHS (2017-2018).

Variables	Attributes	*Stunting*	*Wasting*	*Underweight*
		Adjusted odd ratio (CI)	Adjusted odd ratio (CI)	Adjusted odd ratio (CI)
*Child characteristics*				
Age of child (months)	0–6	—	—	—
	7–12	0.912 (0.568–1.467) 0.705	1.051 (0.584–1.892) 0.869	0.861 (0.530–1.399) 0.546
	13–24	2.481^*∗*^ (1.714–3.593)	0.557 (0.299–1.036) 0.065	0.854 (0.562–1.295) 0.457
	25–36	3.823^*∗*^ (2.637–5.543)	0.436 (0.289–1.026) 0.076	1.128 (0.762–1.672) 0.548
	37–48	3.435^*∗*^ (2.306–5.114) <	0.543 (0.244–1.087) 0.287	1.100 (0.7412–1.630) 0.636
	49–60	3.194^*∗*^ (2.049–4.978)	0.894 (0.447–2.030) 0.792	0.862 (0.567–1.311) 0.487

Child sex	Male	—	—	—
	Female	0.751^*∗*^ (0.608–0.929)	1.016 (0.621–1.662) 0.949	0.853 (0.679–1.071) 0.172

Child birth size	Small (<45.7 cm)	—	—	—
	Average (45.7–60 cm)	0.489^*∗*^ (0.381–0.628)	1.291 (0.720–2.317) 0.391	0.538^*∗*^ (0.413–0.701)
	Large (>60 cm)	0.288^*∗*^ (0.170–0.486)	0.161 (0.021–1.237) 0.079	0.286^*∗*^ (0.152–0.537)

Breastfeeding initiation	Instantly	—	—	—
	Within 1^st^ hour	0.956 (0.456–2.011) 0.916	0.965 (0.166–5.378) 0.967	1.761 (0.355–8.574) 0.468
	Within 1^st^ day	0.828 (0.500–1.370) 0.478	0.774 (0.260–2.261) 0.644	1.063 (0.408–2.744) 0.874

Breastfed	No	—	—	—
	Yes	1.132 (0.985–1.766) 0.064	0.919 (0.448–1.887) 0.818	1.087 (0.333–3.547) 0.891

Postnatal checkup	No	—	—	—
	Yes	1.115 (0.843–1.421) 0.440	1.018 (0.334–2.942) 0.979	1.121 (0.717–1.800) 0.617

Fresh milk consumption	No	—	—	—
	Yes	1.025 (0.438–2.381) 0.926	0.514^*∗∗*^ (0.319–0.879)	0.994 (0.361–2.742) 0.991

Consumed formula milk	No	—	—	—
	Yes	0.828 (0.214–3.429) 0.816	0.727 (0.315–1.676) 0.436	0.986 (0.569–1.705) 0.979

Birth order number	1^st^ born	—	—	—
	2^nd^–4^th^	1.078 (0.445–2.611) 0.859	0.978 (0.537–1.774) 0.958	1.021 (0.757–1.377) 0.829
	>5	1.217 (0.440–3.224) 0.709	0.876 (0.421–1.827) 0.730	1.029 (0.737–1.435) 0.810

*Maternal characteristics*				
Mother's age	15–19	—	—	—
	20–34	1.921 (0.504–7.319) 0.339	1.597 (0.510–4.999) 0.421	1.022 (0.541–1.931) 0.945
	35–49	0.890 (0.188–4.206) 0.883	1.147 (0.274–4.803) 0.851	0.777 (0.379–1.593) 0.491

Education of mother	No education	—	—	—
	Primary	0.733^*∗∗*^ (0.516–1.039)	1.162 (0.537–2.517) 0.703	0.704 (0.472–1.049) 0.085
	Secondary and higher	0.584^*∗*^ (0.424–0.806)	0.953 (0.447–2.030) 0.900	0.668^*∗∗*^ (0.459–0.973)

Maternal BMI	Underweight	—	—	—
	Normal	0.940 (0.646–1.369) 0.747	1.304 (0.578–2.947) 0.524	0.721 (0.514–1.012) 0.058
	Overweight and obese	0.724 (0.488–1.076) 0.110	1.391 (0.599–3.229) 0.443	0.585^*∗∗*^ (0.403–0.850)

Mother's employment	Not employed	—	—	—
	Employed	0.880 (0.344–2.213) 0.813	0.277^*∗∗*^ (0.114–0.781)	1.162 (0.853–1.582) 0.341

Cigarette smoking	No	—	—	—
	Yes	1.098 (0.629–1.017) 0.743	1.100 (0.336–3.603) 0.875	0.938 (0.299–2.942) 0.912

Iron tablets' consumption during pregnancy	No	—	—	—
	Yes	0.785^*∗∗*^ (0.627–0.982)	1.105 (0.668–1.828) 0.697	1.025 (0.467–2.250) 0.951

*Socioeconomics characteristics*				
Geographical area	Punjab	—	—	—
	Sindh	2.231^*∗*^ (1.570–3.170)	1.477 (0.614–3.550) 0.384	3.349^*∗*^ (2.275–4.929)
	KPK	1.120 (0.760–1.652) 0.567	1.572 (0.649–3.809) 0.317	0.846 (0.508–1.408) 0.520
	Baluchistan	1.777^*∗*^ (1.212–2.604)	2.176 (0.876–5.404) 0.094	3.454^*∗*^ (2.288–5.214)
	GB	1.401 (0.860–2.279) 0.153	0.226 (0.016–1.781) 0.146	0.679 (0.331–1.383) 0.310
	ICT	0.677 (0.328–1.389) 0.311	1.237 (0.308–4.729) 0.757	0.687 (0.259–1.800) 0.449
	AJK	0.818 (0.516–1.347) 0.445	0.466 (0.112–1.848) 0.275	0.716 (0.371–1.316) 0.257
	FATA	2.147^*∗*^ (1.416–3.318)	1.4160 (0.5139–3.9017) 0.501	1.569 (0.950–2.593) 0.079

Type of residence	Urbanicity	—	—	—
	Rurality	1.004 (0.770–1.307) 0.909	1.023 (0.571–1.825) 0.902	0.781 (0.585–1.041) 0.115

Water source	Unimproved	—	—	—
	Improved	0.73^*∗∗*^ (0.543–0.962)	0.801 (0.473–1.359) 0.411	0.645^*∗*^ (0.484–0.860)

Father's education	No education	—	—	—
	Primary	0.917 (0.641–1.252) 0.553	0.745 (0.320–1.715) 0.514	0.541 (0.275–1.054) 0.066
	Secondary and higher	0.813 (0.621–1.074) 0.159	1.001 (0.540–1.175) 0.62	0.870 (0.519–1.454) 0.613

Toilet facility	No facility	—	—	—
	Not improved	1.121 (0.750–1.675) 0.534	0.875 (0.312–2.423) 0.803	0.700 (0.465–1.024) 0.064
	Improved	1.036 (0.723–1.428) 0.870	1.965 (0.871–4.421) 0.089	0.770 (0.561–1.057) 0.110

Economic status	Poor	—	—	—
	Middle	0.559^*∗*^ (0.403–0.775)	0.351^*∗*^ (0.172–0.716)	0.462^*∗*^ (0.317–0.627)
	Rich	0.545^*∗*^ (0.414–0.753)	0.521^*∗*^ (0.317–0.855)	0.398^*∗*^ (0.271–0.583)

^*∗*^base category, CI, confidence interval; KPK, Khyber Pakhtunkhwa; GB, Gilgit Baltistan; ICT, Islamabad Capital Territory; AJK, Azad Jammu and Kashmir; FATA, Federally Administered Tribal Area. ^*∗*^indicates significant value at less than 1% level of significance; ^*∗∗*^indicates a significant value at less than 5% level of significance.

## Data Availability

The quantitative data used to support the findings of this study are available from the corresponding author upon request.
